# Gender differences in long-term mortality after spontaneous intracerebral hemorrhage in southern Portugal

**DOI:** 10.1097/j.pbj.0000000000000137

**Published:** 2021-08-04

**Authors:** Joana Teles, Joana Martinez, Maria Mouzinho, Patrícia Guilherme, Ana Marreiros, Hipólito Nzwalo

**Affiliations:** a Faculty of Medicine and Biomedical Sciences, University of Algarve, Faro; b Neurology Department, Centro Hospitalar Universitário do Algarve; c Algarve Biomedical Center, Algarve, Portugal.

**Keywords:** gender, intracerebral hemorrhage, long-term mortality, stroke

## Abstract

**Introduction:**

the prognosis of spontaneous intracerebral hemorrhage (SICH) remains poor. Understanding gender differences can clarify the clinico-epidemiological and process of care related factors that influence SICH prognosis. We analyzed the long-term gender differences of mortality after SICH in Algarve, southern Portugal.

**Patients and Methods:**

analysis of consecutive community representative of SICH survivors (2009–2015). Logistic regression analysis and Kaplan–Meier method was used to assess gender differences on 1-year mortality and survival. We further analyzed if differences exist between 4 age and gender based subgroups (women <75 years, women ≥75 years, men <75 years, men ≥75 years).

**Results:**

a total of 285 survivors were analyzed; majority men (66.3%). Women were 2 years older on average. Overall case fatality was 11.6% [CI: 8.3–15.8]. A non-statistically significant (*P* = .094) higher case-fatality rate was observed in women; men were more frequently admitted to stroke unit; women had more often poor functional outcome or modified Rankin scale (mRS) ≥3. Predictors of death were: being women with ≥ 75 years, in-hospital pneumonia and hospital discharge mRS ≥3. The likelihood of death was higher in women ≥75 years (OR = 2.91 [1.23–8.1], *P* = .035) in comparison to women <75 years and men ≥75 years. Women <75 years had the longest survivor time, whereas women ≥75 years the shortest survivor time (*P* < .001).

**Conclusion:**

gender and age interact to influence long-term mortality after SICH. Women ≥75 years are at increased risk of death and have reduced survival after SICH in southern Portugal. Further studies are needed to clarify the biological or social factors contributing for the poor prognosis in the very old women in the region.

## Introduction

In contrast to acute ischemic stroke, the short and long-term prognosis of spontaneous intracerebral hemorrhage (SICH) remains poor.^1^ At the time of first ever SICH, women are older, have more often poor functional status and better cerebrovascular risk profile.^2–6^ There is evidence showing that gender may mediate ischemic stroke outcomes through biological and non-biological factors.^7^ Data on gender differences on SICH prognosis is inconsistent, sparse and limited to the short-term analysis. For instance, in some studies women were found to have worse^8,9^ while in other better^4^ or similar outcomes^2,3,5,10^ after SICH. Furthermore, the acute phase gender differences of prognosis may vanish on the long-term.^11,12^ Differences in methodology, population characteristics, or specificities of SICH process of care may explain the inconsistencies found.^13^ The study of gender differences can provide insights on the contribution of clinico-epidemiological, and process of care related factors influencing SICH prognosis. Therefore, we sought to investigate the existence of long-term gender differences of mortality in a community representative cohort of SICH in Algarve, southern Portugal.

## Materials and methods

This study was based on a consecutive adult (≥18 years) short-term (30-day) survivors from SICH from Algarve (2009–2015). Details of case identification and data collection are described elsewhere.^14^ Briefly, the original cohort consisted of 549 consecutive patients from the region and excluded secondary ICH (traumatic, structural lesions and hemorrhagic transformation). For this study we additionally excluded short-term survivors discharged to palliative care. Socio-demographic (gender, age) cerebrovascular risk factors (unhealthy alcohol use, diabetes mellitus); admission clinico-radiological factors (type of hematoma, severity of the ICH assessed with the ICH score,^15^ process of care (time from SICH onset to hospital admission, stroke unit (SU) care, selected complications—hyperactive delirium and pneumonia), prior to admission previous hospitalizations as an indirect indicator of pre-existent diminished physiological function or frailty,^16,17^ discharge destination and neurological functional status (modified Rankin scale, mRS) were extracted. The long-term (365 days) outcome (vital status) was extracted from the centrally updated individual online electronic patient record (National Platform for Health Data).

Student *t* test, chi-square test, and Mann–Whitney *U*-test were used as appropriate for univariate analysis. Logistic regression analysis was used to assess the contribution of gender as an independent predictor long-term mortality. Gender based comparison of survivor time was analyzed with Kaplan–Meier method and log-rank tests. Based on the findings from previous studies showing that age interacts with gender for the prognosis in SICH,^5,18^ we also evaluated if specific age and gender based subgroups (women <75 years, women ≥75 years, men <75 years, men ≥75 years) were associated with the long-term outcome. The cutoff age used was determined for the purpose of comparability with similar studies.^5,18^ A 2-sided *P* value of <.05 was considered significant. All analyses used Stata version 12.0.

The Institutional Ethical Board approved the study. Permission from the National Data Protection Commission was obtained.

## Results

**Figure 1:**
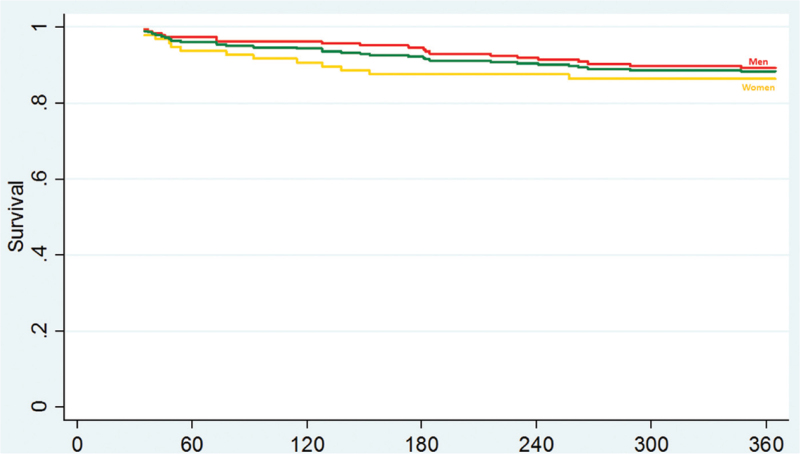
Kaplan–Meier curves show survival after spontaneous intracerebral hemorrhage according to gender. There was no significant difference in the seizure event curves between treatment group (log-rank test *P* = .52).

**Figure 2:**
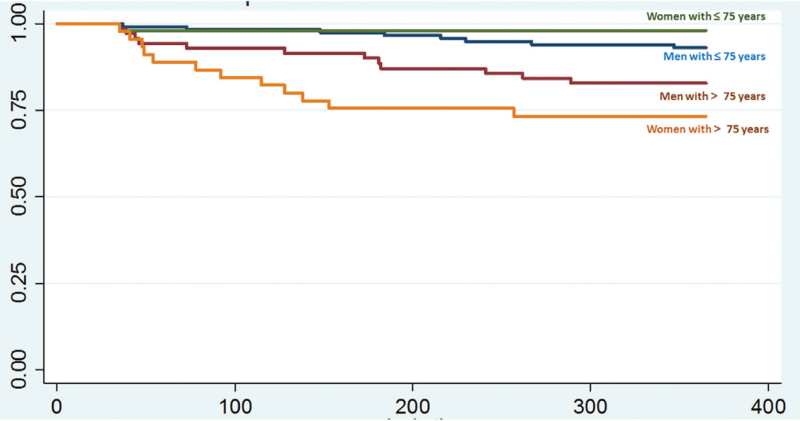
Kaplan–Meier curves show survival after spontaneous intracerebral hemorrhage according to age and gender subgroup. There was a significant difference in death curves between the group (log-rank test *P* < .001).

We identified 360 short-term (30-day) survivors during the study period. Of this group, 71 (19.7%) were in palliative care and 4 (1.1%) were lost to follow-up, leaving a total of 285 SICH survivors to be included in the study. The majority of survivors were men (189/66.3%), and women were approximately 2 years older (72.1 vs 69.2 years, *P* = .04). The overall 1-year case fatality was 11.6% [CI: 8.3–15.8]. A non-statistically significant (*P* = .094) higher case-fatality rate was observed in women in comparison to men, 13.5 [CI: 8.0–21.8] vs 10.6% [CI: 6.9–15.7]. Table 1 resumes the comparison of age groups, risk factors, prior history of hospitalizations, admission clinico-radiological and process of care characteristics. Men had more often history of unhealthy alcohol use (28.6% vs 6.3%, *P* < .001), less often lobar SICH (18% vs 35.4%, *P* = .001). There was no gender difference in the distribution of admission clinical severity assessed by the ICH score. Women were more often admitted ≥6 hours after stroke onset (53.8% vs 49.7%) but the difference was not statistically significant. Men received more often SU treatment (89.4% vs 68.8, *P* = .028) and women had worse discharge neurological functional outcome (82.3% vs 70.4%, *P* = .029). Although no statistically significant, in comparison to men, women were less often discharged to intensive rehabilitation unit (19.8% vs 27.5%). On multivariate analysis, being ≥ 75 years, history of in-hospital pneumonia and discharge poor functional status were predictors of death and female gender did not emerge as an independent factor of outcome (model 1, Table 2). The likelihood of long-term death increased in women with ≥75 years (OR = 2.91 [1.23–8.1], *P* = .035) in comparison to younger women (<75 years) and men ≥75 years (model 2, Table 3). The Kaplan–Meier analysis did not demonstrate gender difference in overall survival rate (Fig. 1), but in the subgroup analysis (Fig. 2) there was a statistically significant difference in the survival rate, with women <75 years having the longest survivor times and women ≥75 years the shortest survivor time (Fig. 2, *P* < .001).

**Table 1:** Gender based comparison of baseline demographics, clinical, imaging and process of care characteristics among 30-days survivors of spontaneous intracerebral hemorrhage in Algarve

	Male (n = 189)	Female (n = 96)	*P* value
Age group, n (%)			.110
<75	119 (63.0%)	51 (53.1%)	
≥75	70 (37.0%)	45 (46.9%)	
Prior to ICH characteristics, n (%)			
Number of previous hospitalizations ≥2, n (%)	30 (15.9%)	13 (13.5%)	.603
Diabetes	50 (26.5%)	27 (28.1%)	.776
Alcohol abuse	54 (28.6%)	6 (6.3%)	**<.001**
Social insertion income	57 (30.60%)	36 (37.50%)	.246
Clinical and radiological admission characteristics, n (%)			
ICH Score			.110
0	92 (48.7%)	33 (34.7%)	
1	68 (36.0%)	42 (44.2%)	
2	25 (13.2%)	14 (14.7%)	
3	4 (2.1%)	4 (4.2%)	
4	0 (0.0%)	1 (1.1%)	
5	0 (0.0%)	1 (1.1%)	
Lobar SICH	34 (18.0%)	34 (35.4%)	**.001**
Admission delay ≤6 h, n (%)	95 (50.3%)	43 (46.2%)	.525
Stroke Unit admission, n (%)	152 (89.4)	66 (68.8)	**.028**
Complications during hospitalization, n (%)			
Pneumonia	26 (13.8%)	14 (14.6%)	.849
Hyperactive delirium	29 (37%)	17 (17.7%)	.380
Discharge modified Rankin scale, n (%)			**.029**
<2	56 (9.6%)	17 (17.7%)	
≥3	133 (70.4%)	79 (82.3%)	
Discharge destination, n (%)			.173
Intensive rehabilitation	52 (27.5%)	19 (19.8%)	
Home	56 (29.6%)	38 (39.6%)	
Nursing/convalescence	81 (42.9%)	39 (40.6%)	

**Table 2:** Multivariate logistic analysis of factors associated with long-term mortality among intracerebral hemorrhage 30-day survivors (model 1)

	Unadjusted OR (95%CI)	*P* value	Adjusted OR (95%CI)	*P* value
Female gender	1.30 (0.62–2.74)	.491	1.20 (0.80–2.32)	.965
≥75 yr	4.63 (2.06–10.39)	<.001	3.58 (1.53–8.39)	**.003**
≥2 hospitalizations prior to SICH	1.96 (0.82–4.68)	.132	1.02 (0.37–2.83)	.969
Lobar SICH	1.21 (0.53–2.74)	.652	0.72 (0.29–1.81)	.486
In-hospital pneumonia	6.69 (2.33–15.66)	<.001	2.12 (1.17–7.33)	**.022**
Social Insertion income	1.96 (0.82–4.7)	.132	2.52 (0.28–6.75)	.075
Stroke Unit admission	0.36 (0.17–0.78)	.009	0.34 (0.11–1.08)	.066
Discharge mRS ≥3	5.36 (2.35–12.22)	.000	3.78 (1.41–10.08)	**.008**
Discharge destination				
Home	1.000 (reference)		1.000 (reference)	
Nursing/convalescence	3.35 (1.28–8.79)	.014	1.64 (0.52–5.15)	.397
Intensive rehabilitation unit	0.46 (0.14–1.58)	.219	0.32 (0.08–1.18)	.087

**Table 3:** Multivariate logistic analysis of factors associated with long-term mortality among spontaneous intracerebral hemorrhage 30-day survivors (model 2)

	Unadjusted OR (95%CI)	*P* value	Adjusted OR (95%CI)	*P* value
Female gender	1.30 (0.62–2.74)	.491	1.20 (0.80–2.32)	.965
Age by gender				
Men <75 yr	1.000 (reference)		1.000 (reference)	
Men ≥75 yr	1.28 (0.44–3.71)	.649	1.86 (0.59–5.86)	.287
Women <75 yr	(0.03–2.19)	.221	0.81 (0.31–2.12)	.671
Women ≥75 yr	2.76 (1.4–6.56)	.042	2.91 (1.23–8.1)	**.035**
≥2 hospitalizations prior to SICH	1.96 (0.82–4.68)	.132	1.05 (0.35–2.81)	.832
In-hospital pneumonia	6.69 (2.33–15.66)	<.001	1.99 (1.11–6.01)	**.022**
Stroke Unit admission	0.36 (0.17–0.78)	.009	0.52 (0.21–1.26)	.147
Discharge mRS ≥3	5.36 (2.35–12.22)	.000	3.21 (1.82–8.91)	**.004**
Discharge destination				
Home	1.000 (reference)		1.000 (reference)	
Nursing/convalescence	3.35 (1.28–8.79)	.014	1.62 (0.54–4.86)	.389
Intensive rehabilitation unit	0.46 (0.14–1.58)	.219	0.36 (0.1–1.29)	.116

mRS = modified Rankin scale; SICH = spontaneous intracerebral hemorrhage.

## Discussion

This is the first study in the region to specifically examine gender differences in the long-term outcome in SICH patients. As in other published studies women were old at the time of stroke, had worse discharge neurological functional status and received less often standard SICH care.^13^ The existence of gender based disparities and the absence of gender based differences in mortality after SICH was demonstrated in the same population and region.^6,14^ As shown by others,^2,3,5,10^ gender in general did not emerged as predictive factor for long-term death and was not associated with decreased survivor time. However, the most important finding was the demonstration of increased risk of death and reduced survivor rate in older women (≥75 years) in comparison to men of all group ages. The discharge functional status was included in the multivariate analysis and was also a predictor long-term outcome. For this reason, the possibility of poor functional status being an explanation for this finding is unlikely. By excluding patients under palliative care we also reduced the impact of active or passive limitation of care which is more problematic for women.^13^ The combined effect of age and gender on the outcome was examined previously by Umeano et al^18^ who demonstrated that older women were at higher risk of being discharge to hospice or death in comparison to men. As in our study, younger women were at lower risk of unfavorable outcome.^18^ Reasons for gender disparities in SICH diseases are complex and involves the interaction of sociodemographic, clinical and process of care factors. Several potential factors may explain the poor long-term outcome after SICH in elderly women. For instance, we had much higher proportion of lobar hemorrhages in women. Lobar SICH is associated with cerebral amyloid angiopathy, increases with age and has higher rates of recurrence.^19^ It is reasonable to admit that age depended factors such as the comorbidity burden,^20^ coexistence and severity of white matter disease^21^ may put elderly women at risk of dying after SICH. Long-term gender disparities in the management of vascular risk factors such as hypertension^22^ or specific management of complications, for instance epilepsy^23^ may also be implicated.

Apart from population specificities or process of care characteristics, methodological differences probably explain the conflicting findings in studies addressing long-term differences in SICH outcomes.^24^ As an example, when studying gender differences on long-term survival after SICH, Zia et al^25^ did not exclude short-term deaths or palliative care patients. It was found that women had better survival than men, but the difference was largely explained by a higher short-term mortality in male ≥75 years. Importantly, as in our study, they found that women <75 years had the longest long-term survival time.

In addition to the limitations inherent to retrospective nature of our study, variables such as comorbidity burden, depression,^20^ presence of white matter disease,^21^ control of vascular risk factors such as hypertension,^22^ socioeconomic deprivation, sociocultural gender roles^13^ that might impact the long-term prognosis were not analyzed.

In conclusion, our study has shown that after controlling for confounding variables such as stroke severity, discharge functional status and destination, women with ≥75 years had decreased survivor time and were at increased risk of dying in the first-year after SICH onset. Further studies are needed to clarify the reasons behind this finding.
